# Cross-sectional and longitudinal analysis of the active commuting behaviors of U.S. Department of the Interior employees

**DOI:** 10.1186/s12889-019-6746-9

**Published:** 2019-05-08

**Authors:** David R. Paul, Yazhuo Deng, Philip S. Cook

**Affiliations:** 1 Department of Movement Sciences, University of Idaho, P.O. Box 442401, Moscow, Idaho 83844-2401 USA; 2 Policy Analysis Group, University of Idaho, 875 Perimeter Drive MS 1134, Moscow, Idaho 83844-1134 USA

**Keywords:** Active commuting, Physical activity, Transportation

## Abstract

**Background:**

Despite evolving evidence of the health and economic benefits of active transportation (AT) to work, few studies have examined the determinants of AT in large organizations with multiple worksites nor how trends in commuting change over time.

**Methods:**

The data were obtained from the U.S. Department of the Interior Employee Commuting Census of 2010 (*n* = 23,230), and 2012–2016 (*n* = 21,725-25,974). The respondents were grouped into four commuting categories: non-active mode, walking, biking, and mixed-mode. Multinomial logistic regression analysis was utilized to examine the correlates of choosing AT to work for the 2010 data. Next, a repeated cross-sectional analysis was completed for all six years of data.

**Results:**

In 2010, AT modes were only chosen by approximately 10% of respondents. Employees who lived farther from work and did not have a public transit station within 0.5 miles from home were generally less likely to choose AT. Respondents working in non-metro workplaces were less likely to bike or take mixed-modes to work, but more likely to walk. Men were more likely to choose AT modes, particularly biking. Respondents aged ≤30 yrs. were less likely to bike than those 31 to 40 yrs., but more likely than those ≥61 yrs. In 2010, the number of respondents that walked was higher, and biked and took mixed-modes was lower when compared to 2016, while the choice to take mixed-modes was higher in 2012 and 2013 when compared to 2016. Daily commuting distances in 2016 tended to be lower than 2010 and 2012, and higher than 2013. However, overall AT choice and commute distance remained reasonably stable over time.

**Conclusions:**

Respondents who lived close to their workplace and a public transportation station, worked in a metro location, were male and younger were more likely to choose AT modes to work. The results provide insight for the U.S. Department of the Interior and other large organizations to develop intervention strategies that support AT to work. Further research is warranted to understand the concurrent individual, social, and environmental barriers and facilitators for choosing AT to work.

## Background

Evidence supporting the promotion of physical activity as a means to reduce the risk of developing non-communicable diseases has become well accepted, and in turn, led to the development of a National Physical Activity Plan [1] and recommendations by the US Department of Health and Human Services [2]. The National Physical Activity Plan [1] recognized that physical activity is influenced by an array of factors that span personal, family, institutional, community, and policy levels. Furthermore, the plan recommended that different societal sectors, such as business and industry promote physical activity in their employees by providing opportunities and incentives for adopting and maintaining a physically active lifestyle.

However, a potential problem with these guidelines is the inability of many Americans to dedicate time in their day to meet them. One alternative to meet recommended levels of physical activity is to promote active transportation (AT; transportation mode that involves moving from place to place by any human-powered transportation mode) to work instead of driving. Individuals that choose AT modes (or a combination of modes) may be more physically active than individuals that choose non-active modes [3, 4]. Costa et al. [5] estimated that approximately half of the generally recommended amount of moderate-to-vigorous physical activity could obtained by AT to work. Active transportation to work has been associated with a reduced likelihood of obesity and cardiovascular disease risk, and higher fitness [6, 7].

Despite the promise of AT as a way for Americans to meet physical activity guidelines, overall participation in AT to work is rather low [2, 8, 9]. A variety of sociodemographic characteristics may be associated with the reasons for not choosing AT to work, such as age [9–15], race/ethnicity [9, 12, 16], education [9, 17], income [9, 14, 15], and sex [9, 11, 12, 15–19]. Also, living in metro (urban) or non-metro (rural) areas may impact the odds of commuting to work by walking, biking, or mixed modes [8, 9], possibly in part due to a lack of infrastructure that would support AT modes [8, 20].

An array of environmental correlates may also impact AT [14, 21, 22]. Macro-level examinations of AT behavior settings suggest that environmental attributes such as population density, accessibility of public transit, and other commuting infrastructure may determine travel behavior [8, 14, 23]. A study focused on commuting in the metro core and inner suburbs of the Washington, DC region found that population density and residence in the metro core area were both positively correlated with the likelihood of choosing AT to work [16]. Yet, questions remain about how this wide range of factors impact AT choice to work in large organizations**,** particularly those with employees working in a variety of metro and non-metro sites. Unfortunately, much of what is known about AT behavior comes from relatively small scale studies from a particular community [10, 20] or large regional/national studies [8, 14].

Factors specific to the location of home relative to work also appear to be significant predictors of choosing AT to work. For example, Cerin et al. [24] reported that distance between work and home was the most important factor for choosing to walk to work. Similarly, Yang et al. [25] found that both walking distance from a transit stop, and distance between home and work were significant predictors of AT. Overall, AT may be appropriate for distances of up to 5 km [20] and that people who live closer to work are more likely to be active commuters [9, 10, 19, 22]. However, it should be noted that variables such as self-reported AT distance may be subject to recall bias [26].

Another aspect of understanding the nature of commuting behavior in organizations is how trends may change over time. Reports from the US Census Bureau [27] indicate that the rates of AT to work are low for biking (0.5–0.6%) and walking (2.0–6.0%), and have changed little over time. The National Household Travel Survey [28] indicated that commuting to work with private vehicles has only ranged from a low of 91.4% to a high of 92.8%, and walking has only ranged from 3.0 to 4.0%. Although changes in AT behavior may be modest, factors that are associated with adopting AT are no children in the household, access to public transportation, convenient access to bicycle routes, and distance living from work [22, 29]. However, changes in the rates of AT of a large organization over time have not been reported despite recommendations that surveillance systems should be in place for workplaces [1].

An additional benefit associated with increasing physical activity by AT is the possibility of reduced health care expenditures [30–33] due to a reduction in the risk of non-communicable diseases such as coronary heart disease, stroke, type 2 diabetes, breast cancer, obesity, hypertension, and colon cancer [12, 34, 35]. The health care expenditures associated with physical inactivity not only impacts individuals, but federal and third party resources (such as health insurance) [34]. The health benefits to individuals also extends to employers, including reduced cost of health care and improved worker performance [1]. Overall, a majority of worksite physical activity interventions result in positive impacts for employees, such as providing organizational and environmental supports for AT [36].

Therefore, the goal of the present investigation was to examine the correlates of AT to work in a large organization within a cross-sectional analysis from a single year of data (2010) and report on changes in the rates of AT by conducting a repeated cross-sectional analysis over time (2010, 2012–2016).

## Methods

### Cross-sectional analysis of 2010 data

The survey of the U.S. Department of the Interior (DOI) employees was done as part of Executive Order 13514-Federal Leadership in Environmental, Energy, and Economic Performance. The DOI partnered with the university to create the comprehensive greenhouse gas emissions (GHG) inventory through an internet-based survey in 2010. The questionnaire was developed based on the *Commuter Choice Survey* [37] created by the U.S. Department of Transportation’s Volpe Center, and was recommended by the Council on Environmental Quality for developing federal agencies’ GHG inventories and reduction strategies. Although the primary purpose of the survey was to create a report that met federal GHG reporting guidelines for use by the DOI, it became apparent that the nature and scope of the data could contribute to the planning and health science fields.

An invitation to participate and hyperlink to the survey were emailed to all permanent-fulltime employees of the DOI. Two additional follow-up email reminders were sent to the remaining employees who had yet to complete the questionnaire. Of the initial 52,963 e-mail invitations sent to permanent-fulltime employees, 2918 (5.5%) were returned with a “user unknown” error, so they were assumed to be invalid. From the remaining valid 50,045 addresses, responses were received from 25,346 employees (response rate of 50.6%). Respondents that did not report a commuting mode for all five days of the survey were deleted (*n* = 2115), resulting in a final sample size of 23,231 (46.4% response rate).

Survey questions asked respondents to recount their commute trips to and from work during the week prior to taking the survey. Respondents could choose to describe the trip for each work day or choose a “same as previous day” answer if their commute trips were the same on consecutive days. Also, respondents could describe their trips to work by choosing up to three segments based on transportation mode choice and distance for each segment. One of 12 pre-defined modes of transportation (Car, Truck/SUV, Van, Federal Vehicle, Motorcycle/Scooter, Transit bus, Transit rail, Commuter rail, Intercity rail, Walk, Bicycle, Ferry boat) or “other”, and a distance for each segment were described. If the commute home was different, the respondent was asked to describe it in the same manner as the commute to work. Distance in miles from home to workplace was reported as an open-ended question. One-way commute time to work was calculated by averaging the duration for commuting to work and commuting to home on an average traffic day. Distance between home and nearest public transit station or bus stop was reported as an 8-item categorical variable, which was reduced six categories (< 0.5, 0.5–1.0, 1.0–1.5, 1.5–2.0, and > 2.0 miles, and “Don’t know”).

### Commuting category definitions

Respondents were assigned to one of six groups based on the modes chosen to describe their commute trips for the sample week. *Non-active commuters* were respondents who chose to drive some type of vehicle (such as a car) to work for all five days of the survey (all segments of each trip). *Walkers* were commuters that walked for at least three commute trips during the sample week, while *bikers* were commuters that biked for at least three commute trips during the sample week. *Mixed-mode* described commuters that walked or biked for one segment in combination with a non-active mode for at least one trip during the sample week. Those who did not commute or chose “other” for all five days of the survey were placed in separate categories.

### Demographic variables (2010 only)

Sex was reported as a categorical variable (male/female). Age was also reported as a categorical variable (18–25, 26–30, 31–35, 36–40, 41–45, 46–50, 51–55, 56–60, 61–65, 66–70 and > 70 yrs), then subsequently collapsed into five categories due to small sample sizes for some of the comparisons (≤30, 31–40, 41–50, 51–60, and ≥ 61 yrs). Respondents were asked to include the ZIP Code for their workplace, which was then associated with a county. USDA Economic Research Service’s Rural Urban Continuum Codes (RUCC) designation for each county was used to determine whether the respondent worked in a metro or non-metro county. Counties with RUCC’s 1–3 were considered metro and 4–9 were considered non-metro [38].

### Longitudinal analysis of 2010, 2012–2016

The yearly administration of the survey continued in 2012 by the General Services Administration until 2016. Starting in 2012, the survey was modified to exclude most of the demographic information, but the rest of the survey was similar. Therefore, we conducted a longitudinal analysis of how commuting behaviors may have changed over the course of the six-year survey by utilizing a repeated cross-sectional analysis approach (since the identities of the respondents could not be tracked over time). The sample sizes were as follows; 2012 (*n* = 25,974), 2013 (*n* = 24,686), 2014 (*n* = 23,078), 2015 (*n* = 21,725), and 2016 (n = 23,609).

### Data analysis

Non-response to the 2010 demographics questions occurred in 0.2 to 1.9% of the cases, so missing responses were imputed with the PROC MI procedure in SAS [39]. Non-response to daily commuting distance for all survey years occurred in approximately 9% of all trips. Also, it was clear that there were obvious unrealistic responses to commuting distance (such as an individual who reported walking 200 miles in a day to work), therefore individuals with reported commuting distances >95th percentile were identified. Commuting trip distances that were missing or unrealistic were imputed with the median trip distance for each commuting category. Respondents that reported missing distance for all five days of the survey were excluded from the analysis (average of 1.7% per year).

Multinomial logistic regression was used to investigate the associations between commute modes and distance between home and work, distance between home and nearest public transit station, metro/non-metro workplace location, sex, and age from the 2010 survey. Since the data from 2010 to 2016 were repeated cross-sectional data, the multinomial logistic regression was performed for commuting modes on discrete time (in years) and metro/non-metro work location [40]. Multilevel linear regression analyses examined associations of daily commuting distances (reported each day in a week) with discrete time (in years) and metro/non-metro work location, using within subject clusters as a random intercept. The multinomial logistic regression models and the random intercept model was fitted using the R packages nnet [41], lme4 [42], and lmerTest [43], respectively. An a priori α of 0.05 was set as the criteria for detecting the statistical significance. Except for the imputation of missing values, all analyses were performed using R, version 3.5.2.

## Results

### Cross sectional analysis of 2010 data

Overall, there were 23,231 respondents with valid responses (Table 1 shows the descriptive statistics of the employees choosing different commuting modes). The largest proportion of the respondents lived less than 0.5 mile of a public transit station (37.2%), worked in metro areas (76.9%), were male (55.0%), and between 51 and 60 years of age (38.5%). Of those, 20,505 (88.3%) chose non-active modes, while 633 walked (2.7%), 632 biked (2.7%), and 1119 chose mixed modes (4.8%). Also, 334 were non-commuters (1.4%) and 7 chose “other” (0.03%). The average distance to work was approximately 15 miles, with an average one-way commute time of 26 min.

**Table 1:** Descriptive statistics for 2010 (n=23,231)

	All	Non-Active	Walk	Bike	Mixed- Mode	Non-Commute	Other
	Mean (SD)	Mean (SD)	Mean (SD)	Mean (SD)	Mean (SD)	Mean (SD)	Mean (SD)
Distance between home and work (miles)							
	14.6 (11.7)	15.5 (11.6)	0.96 (0.7)	4.0 (2.7)	13.9 (10.5)	13.0 (11.1)	19.7 (23.0)
Commute time (minutes)							
	26.0 (16.0)	26.0 (15.0)	7.1 (5.7)	16.1 (8.7)	43.7 (22.2)	23.2 (16.0)	19.1 (3.8)
Distance between home and nearest public transit station (miles)							
	n (%)	n (%)	n (%)	n (%)	n (%)	n (%)	n (%)
<0.5	6,327 (37.2)	5,011 (34.1)	271 (60.8)	367 (68.6)	580 (54.3)	94 (37.3)	4 (80.0)
0.5-1.0	2,028 (11.9)	1,753 (11.9)	40 (9.0)	65 (12.1)	139 (13.0)	31 (12.3)	0 (0.0)
1-1.5	1,233 (7.3)	1,101 (7.5)	19 (4.3)	27 (5.0)	77 (7.2)	9 (3.6)	0 (0.0)
1.5-2.0	964 (5.7)	876 (6.0)	5 (1.1)	13 (2.4)	59 (5.5)	11 (4.4)	0 (0.0)
>2	4,728 (27.8)	4,335 (29.5)	84 (18.8)	39 (7.3)	195 (18.2)	74 (29.4)	1 (20.0)
Don't know	1,708 (10.1)	1,605 (10.9)	27 (6.1)	24 (4.5)	19 (1.8)	33 (13.1)	0 (0.0)
Work location							
Metro	17,853 (76.9)	15,674 (76.4)	363 (57.3)	517 (81.8)	1,042 (93.1)	252 (75.4)	5 (71.4)
Non-metro	5,377 (23.1)	4,831 (23.6)	270 (42.6)	115 (18.2)	77 (6.9)	82 (24.6)	2 (28.6)
Sex							
Male	12,766 (55.0)	11,046 (53.9)	406 (64.1)	503 (79.6)	635 (56.7)	172 (51.5)	4 (57.1)
Female	10,464 (45.0)	9,459 (46.1)	227 (35.9)	129 (20.4)	484 (43.2)	162 (48.5)	3 (42.9)
Age (years)							
≤30	1,403 (6.0)	1,237 (6.0)	48 (7.6)	39 (6.2)	63 (5.6)	16 (4.8)	0 (0)
31 to 40	3,876 (16.7)	3,380 (16.5)	135 (21.3)	138 (21.8)	177 (15.8)	46 (13.8)	0 (0)
41 to 50	6,564 (28.3)	5,847 (28.5)	168 (26.5)	173 (27.4)	305 (27.3)	68 (20.4)	3 (42.9)
51 to 60	8,942 (38.5)	7,857 (38.3)	222 (35.1)	240 (38.0)	458 (40.9)	163 (48.8)	2 (28.6)
≥61	2,445 (10.5)	2,184 (10.6)	60 (9.5)	42 (6.6)	116 (10.4)	41 (12.3)	2 (28.6)

*SD* standard deviation

The multinomial logistic regression analysis of the 2010 survey indicated that distance between home and work, distance between home and nearest public transit station, workplace location, sex, and age were all significant predictors of choosing between AT and non-active modes (Table 2). Overall, respondents who reported longer distances between workplace and home were less likely to choose walking (AOR = 0.609, *p* < 0.001) and biking (AOR = 0.824, *p* < 0.001) to work when compared to non-active modes. Having a public transit station located greater than 0.5 mile from home tended to decrease the likelihood of choosing AT over non-active modes. Workplaces located in non-metro areas were a significant disincentive for individuals to choose biking (AOR = 0.689, *p* = 0.005) and mixed-modes (AOR = 0.540, p < 0.001), whereas respondents in non-metro workplaces were more likely to walk to work (AOR = 1.276, *p* = 0.032) over non-active modes. In addition, age was a significant determining factor for biking to work as 31–40 yr. olds were more likely and ≥ 61 yr. olds less likely to bike to work when compared to the youngest age category (≤30 yr. olds). Finally, male employees showed a higher likelihood of choosing all AT modes over non-active modes than did their female counterparts.

**Table 2:** Multinomial logistic regression analysis for 2010 (n=23,231)

			Commuting Mode^a^			
	Walk		Bike		Mixed-Mode	
	AOR	*p*-value	AOR	*p*-value	AOR	*p*-value
Distance between home and work (miles)						
	0.609	<0.001	0.824	<0.0001	1.004	0.1082
Distance between home and nearest public transit station (miles)						
<0.5	Reference		Reference		Reference	
0.5-1.0	0.582	0.003	0.639	0.002	0.688	0.001
1.0-1.5	0.551	0.018	0.518	0.002	0.593	<0.001
1.5-2.0	0.372	0.013	0.434	0.004	0.564	0.001
>2.0	1.019	0.902	0.351	<0.001	0.382	<0.001
Don't know	0.449	0.001	0.313	<0.001	0.105	<0.001
Not available	0.561	<0.001	0.360	<0.001	0.092	<0.001
Work location						
Metro	Reference		Reference		Reference	
Non-metro	1.276	0.032	0.689	0.005	0.540	<0.001
Sex						
Female	Reference		Reference		Reference	
Male	1.559	<0.001	3.968	<0.001	1.186	<0.001
Age (years)						
≤30	Reference		Reference		Reference	
31-40	1.233	0.260	1.444	0.043	1.084	0.597
41-50	0.972	0.873	1.092	0.632	1.099	0.513
51-60	0.871	0.428	0.947	0.762	1.193	0.208
≥61	0.742	0.157	0.464	0.001	1.028	0.864

*Non-active commuters* were respondents who chose to drive some type of vehicle (such as a car) to work for all five days of the survey

*Walkers* were commuters that walked for at least three commute trips during the sample week

*Bikers* were commuters that biked for at least three commute trips during the sample week

*Mixed-mode* described commuters that walked or biked in combination with a non-active mode for at least one trip during the sample week

*AOR* Adjusted Odds Ratio

^a^Non-active mode was the reference category

### Longitudinal analysis of 2010, 2012–16

Trends in commuting across the five years of data can be observed in Tables 3, 4, 5. The category of commuters (Table 3) remained reasonably stable, with an overwhelming majority choosing non-active modes (85–88%). After 2010, non-metro commuters increased their mixed-mode selection. Looking at the individual days of commuting (Table 4), the trends were also stable, although the choice of mixed-modes tended to increase after 2010. In Table 5, commuting distances tended to remain stable, although the distances covered during mixed modes tended to decrease after 2010.

**Table 3:** Distribution of commuters

	All (%)				Metro (%)				Non- Metro (%)			
Year	Non-Active	Walk	Bike	Mixed Mode	Non-Active	Walk	Bike	Mixed Mode	Non-Active	Walk	Bike	Mixed- Mode
2010	88.3	2.7	2.7	4.8	87.8	2.0	2.9	5.8	89.9	5.0	2.1	1.4
2012	86.2	2.2	2.9	7.6	86.7	1.5	3.1	7.7	84.9	4.5	2.3	7.4
2013	85.2	2.3	3.5	7.9	85.6	1.6	3.6	8.1	84.1	4.7	3.0	7.3
2014	85.8	2.2	3.3	7.4	86.0	1.5	3.5	7.7	85.3	4.3	2.7	6.6
2015	85.5	2.2	3.2	7.5	85.8	1.4	3.4	7.7	84.4	4.9	2.5	6.8
2016	85.8	2.4	3.2	7.0	86.1	1.6	3.4	7.2	84.8	5.0	2.5	6.2

**Table 4:** Distribution of individual days of commuting

	All (%)				Metro (%)				Non- Metro (%)			
Year	Non-Active	Walk	Bike	Mixed Mode	Non-Active	Walk	Bike	Mixed Mode	Non-Active	Walk	Bike	Mixed- Mode
2010	79.9	2.3	2.2	3.7	79.3	1.7	2.3	4.6	82.2	4.3	1.9	0.8
2012	83.5	2.1	2.2	6.1	83.5	1.4	2.3	6.1	83.5	4.2	1.7	6.1
2013	82.1	2.2	2.6	6.2	81.9	1.4	2.8	6.3	82.5	4.5	2.1	5.9
2014	81.6	2.0	2.6	6.0	81.1	1.3	2.8	6.1	82.9	4.2	2.2	5.4
2015	80.9	2.0	2.6	6.0	80.5	1.3	2.7	6.1	82.2	4.4	2.2	5.8
2016	80.4	2.2	2.5	5.7	79.9	1.4	2.7	5.8	82.1	4.6	2.0	5.3

*Non-active commuters* were respondents who chose to drive some type of vehicle (such as a car) to work for all five days of the survey

*Walkers* were commuters that walked for at least three commute trips during the sample week

*Bikers* were commuters that biked for at least three commute trips during the sample week

*Mixed-mode* described commuters that walked or biked in combination with a non-active mode for at least one trip during the sample week

The sample sizes were: 2010 (n=23,231), 2012 (n=25,974), 2013 (n=24,686), 2014 (n=23,078), 2015 (n=21,725), and 2016 (n=23,609)

**Table 5:** Commuting distances by mode and location

	Non-Active		Walk		Bike		Mixed-Mode	
Year	Mean (SD)	Median	Mean (SD)	Median	Mean (SD)	Median	Mean (SD)	Median
All (miles)								
2010	21.2 (23.0)	15	1.21 (7.03)	0.5	4.94 (4.38)	4	18.4 (17.5)	14
2012	22.8 (26.7)	15	0.94 (3.17)	0.5	5.14 (4.39)	4	13.3 (17.0)	8
2013	19.4 (21.3)	13	0.84 (1.28)	0.5	4.65 (3.92)	4	12.1 (13.6)	8
2014	19.9 (21.7)	13	0.85 (0.91)	0.5	4.58 (3.86)	3	12.6 (17.3)	8
2015	20.1 (22.0)	13	0.85 (0.97)	0.5	4.53 (4.10)	3	12.5 (14.4)	8
2016	19.9 (21.9)	13	0.80 (0.79)	0.5	4.43 (3.96)	3	12.3 (13.8)	8
Metro (miles)								
2010	21.3 (22.0)	15	1.28 (2.78)	0.5	5.45 (4.55)	4	18.4 (15.8)	14
2012	22.3 (24.8)	15	1.23 (4.37)	0.5	5.60 (4.40)	5	15.0 (17.6)	10
2013	19.3 (20.4)	13	1.01 (1.49)	0.5	5.10 (3.94)	4	13.9 (14.0)	10
2014	19.6 (20.5)	13	1.01 (0.95)	0.5	5.00 (3.80)	4	14.0 (15.0)	10
2015	19.6 (20.2)	13	1.08 (1.27)	0.5	5.00 (4.14)	4	14.0 (13.7)	10
2016	19.6 (20.9)	13	0.96 (0.87)	0.5	4.90 (4.01)	4	13.9 (13.9)	10
Non-Metro (miles)								
2010	20.9 (25.7)	15	1.14 (10.2)	0.5	2.85 (2.70)	2	18.4 (36.8)	7
2012	24.3 (32.0)	14	0.63 (0.60)	0.5	3.13 (3.76)	2	7.9 (13.4)	4
2013	19.7 (24.1)	12	0.68 (1.01)	0.5	2.92 (3.33)	2	6.0 (9.8)	3
2014	21.0 (25.0)	12	0.69 (0.82)	0.5	3.03 (3.75)	2	7.4 (23.1)	3
2015	21.7 (26.8)	13	0.61 (0.33)	0.5	2.77 (3.44)	2	6.8 (15.4)	3
2016	20.6 (24.9)	12	0.64 (0.66)	0.5	2.49 (2.77)	2	6.8 (12.0)	3

Mean (Standard Deviation)

*Non-active commuters* were respondents who chose to drive some type of vehicle (such as a car) to work for all five days of the survey

*Walkers* were commuters that walked for at least three commute trips during the sample week

*Bikers* were commuters that biked for at least three commute trips during the sample week

*Mixed-mode* described commuters that walked or biked in combination with a non-active mode for at least one trip during the sample week

The sample sizes were: 2010 (n=23,231), 2012 (n=25,974), 2013 (n=24,686), 2014 (n=23,078), 2015 (n=21,725), and 2016 (n=23,609)

The repeated cross-sectional analyses in Table 6 showed that respondents had a higher likelihood to walk to work and lower likelihood to choose biking and mixed commuting mode instead of non-active modes in 2010 than did in other years. In addition, using non-active as the reference, there was a tendency for non-metro commuters to be more likely to walk to work (AOR = 2.996, *p* < 0.001), but less likely to bike (AOR = 0.770, *p* < 0.001) and take mixed-modes (AOR = 0.821, *p* < 0.001) than metro dwellers. The multilevel model with significant random effects (Table 7) reported greater daily commuting distances in 2010 (β = 1.170, *p* < 0.001) and 2012 (β = 2.508, *p* < 0.001) than 2016. After controlling for the years, overall daily commuting distances for non-metro employees were slightly greater (β = 0.466, *p* = 0.001) than those who worked in metro areas, and all AT modes (β_walk_ = − 18.433, β_bike_ = − 4.600, β_mixed_ = − 1.021, all p < 0.001) were associated with shorter daily commuting distances than non-active modes.

**Table 6:** Multinomial logistic regression for distribution of commuters

	Commuting Mode^a^					
	Walk		Bike		Mixed-Mode	
	AOR	*p*-value	AOR	*p*-value	AOR	*p*-value
Year						
2010	1.129	0.040	0.825	<0.001	0.668	<0.001
2012	0.923	0.183	0.906	0.058	1.081	0.026
2013	0.978	0.712	1.092	0.084	1.135	<0.001
2014	0.914	0.148	1.033	0.541	1.058	0.115
2015	0.951	0.424	1.014	0.798	1.072	0.056
2016	Reference		Reference		Reference	
Work location						
Non-metro	2.996	<0.001	0.770	<0.001	0.821	<0.001
Metro	Reference		Reference		Reference	

*Non-active commuters* were respondents who chose to drive some type of vehicle (such as a car) to work for all five days of the survey

*Walkers* were commuters that walked for at least three commute trips during the sample week

*Bikers* were commuters that biked for at least three commute trips during the sample week

*Mixed-mode* described commuters that walked or biked in combination with a non-active mode for at least one trip during the sample week

The sample sizes were: 2010 (n=23,231), 2012 (n=25,974), 2013 (n=24,686), 2014 (n=23,078), 2015 (n=21,725), and 2016 (n=23,609)

*AOR* Adjusted Odds Ratio

^a^Non-active mode was the reference category

**Table 7:** Multilevel model with random intercept effects for daily commuting distances

	β	*p*-value
Constant	19.704	<0.001
Year		
2010	1.170	<0.001
2012	2.508	<0.001
2013	−0.643	0.002
2014	-0.063	0.756
2015	0.082	0.689
2016	Reference	
Work location		
Non-metro	0.466	0.001
Metro	Reference	
Commuting mode		
Walk	−18.433	<0.001
Bike	−4.600	<0.001
Mixed-mode	−1.021	<0.001
Non-active	Reference	

*Non-active commuters* were respondents who chose to drive some type of vehicle (such as a car) to work for all five days of the survey

*Walkers* were commuters that walked for at least three commute trips during the sample week

*Bikers* were commuters that biked for at least three commute trips during the sample week

*Mixed-mode* described commuters that walked or biked in combination with a non-active mode for at least one trip during the sample week

## Discussion

The results of this study suggest a significant association between a range of factors related to the daily commute and AT mode choice to work. Distance between home and work, distance between home and nearest public transition station, workplace location (metro vs. non-metro), sex, and age were related to choosing AT modes. For example, commute distance to work was a significant negative determinant of being an active commuter, which is consistent with previous research [10, 22, 24]. These findings are supported by Panter and colleagues [17] who reported that if residents lived close enough to their workplace, they were more likely to commute by walking or biking. The number of respondents that chose AT modes was reasonably stable over time, although there was a tendency for the choice of mixed-modes to increase. Non-metro respondents were more likely to walk to work, but less likely to bike or choose mixed-modes. Finally, commuting distances tended to decrease over time, were higher in non-metro dwellers, and were lower for AT modes.

The results of this investigation confirm that there are differences between the AT choices of metro and non-metro respondents, whereby employees that worked in metro areas were more likely to bike and take mixed modes to work [8]. However, non-metro respondents were more likely to walk to work, which is inconsistent with Quinn et al. [9], who reported that metro dwellers were more likely to both walk and bike to work. A work-related AT study in the Washington DC region suggested that bikeway infrastructure was significantly related to higher rates of bicycling [16]. At the same time, safe route environments and presence of green space are perceived as important environmental determinants of bicycle commuting [44]. Therefore, a potential exists to develop infrastructure to encourage non-metro commuters to choose bicycling to work, although physical activity choices may also play a role. For example, other research has demonstrated that non-metro residents may engage in less moderate-to-vigorous physical activity than their metro counterparts [45], suggesting that there are other differences between metro and non-metro residents that may impact choosing AT to work. Therefore, further research is warranted to explore environmental and social barriers of AT among non-metro residents.

Understanding demographic influences such as sex and age on AT is critical for developing tailored strategies that encourage AT to work. Consistent with prior investigations [15, 46, 47], our study found that men were more likely to choose AT modes than women, and the oldest respondents were less likely to bike to work than the youngest age group. The likelihood of walking to work was higher in men (OR = 1.559, *p* < 0.0001), but the difference between men and women was particularly strong for bike commuting (OR = 3.968, *p* < 0.0001). Overall, previous research has indicated that bicycle commuting is more popular among men [9, 19, 46, 48, 49], and walking more popular among women [17, 48, 49]. Another explanation for fewer female active commuters is that women may bear the responsibility for day-to-day household tasks, such as child care and housework [48]. The influence of age has also been reported by Quinn et al. [9], whereby they found that walking and bicycling to work decreased with increasing age. Based on the current analysis, we speculate that the age effect on bicycling may be related to safety concerns among older individuals [13]. Future studies should direct attention to the study of demographic differences for AT to understand the challenges for specific subgroups, especially women and older adults.

One of the unique aspects of this study was the longitudinal analysis, perhaps the most comprehensive of its kind. Similar to the present results, Panter et al. [29] reported small changes in walking (+ 3 min/wk) or bicycling (− 5.3 min/wk) commuting over the course of a year. In a follow-up study over the course of seven years, Yang et al. [22] reported that 76% of commuters chose non-active modes and tended to not change their mode, while 8% switched to an active mode. Based on a report from the US Census Bureau [27], the percentage of workers that bicycled to work has only varied between 0.5% in 1980 to 0.6% in the 2008–2012 American Community Survey. In the same report, the percentage of walkers was 5.6% in 1980, which declined to 2.9% in 2000 and remained similar (2.8%) to the 2008–2012 American Community Survey. The National Household Travel Survey [28] indicates that driving a private vehicle to and from work ranged from a high of 92.8% in 1995 to 91.4% in 2009, while walking reached a high of 4.0% in 1990 to 3.0% in 2009 (low of 2.3% in 1995). Therefore, it appears as though AT behaviors have remained relatively constant over time.

Social and cultural supports provided by employers, although not tested in our model, may pose a substantial influence on choice of AT to the workplace. Both governmental agencies and employers could foster an AT culture by increasing awareness of the benefits of AT and offering incentives to promote AT behaviors [25]. For example, employees could be incentivized by programs that provide tax breaks, health insurance premium reductions, public transportation discount/refunds, and other financial incentives [16]. A large organization having multiple worksites in metro and non-metro areas could provide extra support for those who work in non-AT-friendly areas. There is evidence of benefits for an organization to incentivize AT, particularly in health-related factors such as obesity, hypertension, diabetes, and physical fitness [6, 12, 35]. Flint et al. [7] reported that commuting by AT is associated with lower body mass index (BMI) in both men and women. A change from non-active to active modes of commuting is associated with a decrease in BMI [50, 51], as is the maintenance of bicycling commuting over time [52]. However, not all longitudinal analyses support the impact of AT on BMI [52]. Overall, it appears as though increases in physical activity associated with AT use may result in improved health outcomes, potentially resulting in cost savings associated with reduced health care expenditures for workers and their employers [33].

Furthermore, joint efforts between public health and urban planning agencies could advocate for additional environmental supports, such as improved walking and biking infrastructure, and expanded public transit systems. A range of correlates are associated with AT, but environmental constraints appear to be particularly important [21]. Supportive transportation environments may increase the chances of employees taking up walking or bicycling to work [29]. Another option is to provide educational materials, which has been shown to increase walking to work, but not bicycling [53].

The main strength of this study is the inclusion of a nationwide sample of respondents from a large federal agency in the US over the course of six years. Furthermore, this study examines differences in AT mode choice to work from respondents with a wide array of personal characteristics and location of where they live and work. Despite these strengths, a number of limitations of this study should be noted. The self-reported measures of AT may be subject to recall bias, therefore measures such as work distance and commute time may be over- or underestimated [26]. The commuting categories chosen for this study could be considered arbitrary. Moreover, important confounders such as AT for non-work purposes, daily physical activity level, home location, and general health status were not controlled for this analysis. Due to the repeated cross-sectional analysis design, individual respondents could not be tracked over the course of the study. Finally, the respondents from our study only included employees from one organization, thus limiting the generalizability of our findings to other organizations.

## Conclusions

The current study represents the largest of its kind to investigate the characteristics of employees that choose active transportation to work, then subsequently track trends over the course of six years. A range of environmental variables related to the location of home and work, and a number of demographic factors were associated with AT to work for US Department of the Interior employees. However, it is worth noting that approximately 88% of DOI employees reported taking non-active modes to and from work, which is similar to the findings from the American Community Survey [27], the National Household Travel Survey [28], and others [20, 25], but higher than similar investigations [7, 12, 22, 35]. Overall, the percentage of respondents that took active modes was low when compared to other investigations [9, 14, 21, 25], and relatively stable over time.

## Abbreviations

AT: Active Transportation
BMI: Body Mass Index
DOI: US Department of the Interior
GHG: Greenhouse Gas Emissions
RUCC: USDA Economic Research Service’s Rural Urban Continuum Codes
